# Effects of social support on cognitive frailty among the older adults in China: mediation of psychological resilience and moderated mediation of education

**DOI:** 10.3389/fnagi.2025.1579485

**Published:** 2025-05-16

**Authors:** Wenjuan Li, Yiwen Ma, Jinli Wei, Shanzheng Wu, Liangliang Cui, Chengchao Zhou

**Affiliations:** ^1^Jinan Center for Disease Control and Prevention, Jinan, Shandong, China; ^2^School of Public Health, Cheeloo College of Medicine, Shandong University, Jinan, Shandong, China; ^3^Jinan Mental Health Center, Jinan, China

**Keywords:** cognitive frailty, social support, psychological resilience, education, moderated mediation

## Abstract

**Objectives:**

Previous research has revealed a relationship between social support and cognitive frailty. However, the underlying mechanisms of this connection have still not been well explored. The study aimed to investigate the effect of social support on cognitive frailty, mediated by psychological resilience and to examine the moderated mediation effect of education.

**Methods:**

A total of 1,758 older adults aged 60 years and above were included in the analysis. A descriptive analysis was conducted to describe the sample characteristics. The moderated mediation models were examined using Mplus 8.3, in which the mediation variables was psychological resilience, and the moderation variable was education.

**Results:**

The prevalence of cognitive frailty among the older adults was 4.3%. Social support had a significant negative predictive effect on the cognitive frailty (*B* = −0.066, *p* < 0.01), the psychological resilience partially mediated the relationship between social support and cognitive frailty (*B* = −0.103, *p* < 0.001). Compared to illiterate, primary school (*B* = −0.184, *p* < 0.05), middle school (*B* = −0.244, *p* < 0.01) and high school or above (*B* = −0.315, *p* < 0.01) could regulate the relationship between social support and psychological resilience in older adults.

**Discussion:**

We present a conceptual model containing the mediated effects of psychological resilience and the moderated effect of education on the relationship between social support and cognitive frailty. We believe this model enhances understanding of these associations and could be instrumental in formulating intervention strategies to mitigate the incidence of cognitive frailty in older adults.

## 1 Introduction

Aging is one of the predominant trends of the current population structure of the world, especially in China. The “2021 National Bulletin on the Development Aging Care” shows that as of the end of 2021, the population of China aged 60 and above was 26.736 million, accounting for 18.9% (Wang, 2022). This proportion will exceed 30% in 2034 and exceed 40% in 2055, entering a super-aged society (Wang, 2022). China’s population aging is gradually characterized by substantial volume, rapid speed, poor foundation, imbalance, and complexity (Xie et al., 2023). With the further intensifying of population aging, a series of elderly health issues continue to emerge, which not only cause a serious burden to individuals and families, but also has a comprehensive, profound, and widespread impact on the entire economic and social development (Du, 2021).

The aging of the population has led to a significant increase in age-related disease. During the elderly stage, the physiological reserve function of multiple systems in the older adults declines rapidly, resulting in a state of homeostatic imbalance or in a frail state. Frailty, a multi-factor clinical syndrome, is closely associated with age (Fried et al., 2001). Its core is heightened vulnerability and decreased ability to withstand stress, which is reversible and dynamic (Xi et al., 2013). Research has shown that physical factors and cognitive abilities are key factors in predicting mortality risk (Brigola et al., 2015), with frailty and cognitive impairment often appearing early in disability and dementia (Fried et al., 2001; Makizako et al., 2015). These conditions may share common mechanisms, accelerating declines in function. The concept of cognitive frailty (CF) proposed in 2013 by an (I.A.N.A./I.A.G.G.) international consensus group (Kelaiditi et al., 2013), defined as the simultaneous presence of both cognitive impairment and physical frailty in non-demented older adults. CF has emerged as a significant focus in geriatrics, providing better predictions of mortality, disability, dementia, and other adverse health outcomes in the older adults (Bu et al., 2021; Feng et al., 2017; Zheng et al., 2020). Therefore, for older adults with CF, early identification and implementation of effective interventions may prevent or slow the progression of their condition, thereby effectively preventing adverse health outcomes such as disability, hospitalization, and mortality.

The manifestation of CF and adverse health outcomes in the older adults is not only related to biological genetics, but also affected by social factors. Social support (SS) refers to various forms of assistance and support from family, friends, communities and social organizations, including emotional support, information exchange and practical help (Kelly et al., 2017). As a psychosocial factor, SS has an important impact on the cognitive health. Sufficient SS can not only provide the emotional care and practical assistance that the older adults need, but also foster positive social interaction and cognitive stimulation, thereby playing a positive role in delaying CF and preventing cognitive function decline (Aarts et al., 2015; Evans et al., 2018; Lara et al., 2019). Psychological resilience (PR) refers to an individual’s ability to adapt and recover in the face of stress and challenges. High levels of PR can enable individuals to maintain or restore normal coping abilities in adversity. Good SS is believed to have a positive impact on an individual’s mental health (Piolatto et al., 2022). Research has shown that SS can significantly improve the PR of older adults (Ozbay et al., 2007). Old adults with high levels of PR tend to sustain better cognitive function compare to those with lower PR levels, thus potentially helping to mitigate the impact of aging on the brain and slow the progression of cognitive vulnerability (Chang et al., 2023; Liao et al., 2022). A robust SS network can fortify an individual’s PR, thus helping to cope with stress and challenges in life. Individuals with high levels of PR show better coping strategies and adaptive abilities when faced with the risk of cognitive decline (Li et al., 2021; Lou et al., 2023). Based on the above analysis, PR may mediate the relationship between SS and CF.

Education generally has a positive relationship with SS. People with higher levels of education are more likely to participate in social activities, obtain rich emotional support, and establish closer social connections, because they have wider social resources and more social relationships (Krause and Shaw, 2000). At the same time, research shows that people with higher levels of education tend to be more capable of problem-solving skills, and greater self-regulation and improved emotional management (Cosco et al., 2018), all of which are closely associated to PR. In addition, education is also associated with the establishment and utilization of SS networks, which also helps individuals better cope with challenges and recover mental health (Hildon et al., 2008; Thoits, 2011). Based on the above analysis, education may moderate the relationship between SS and PR.

**FIGURE 1:**
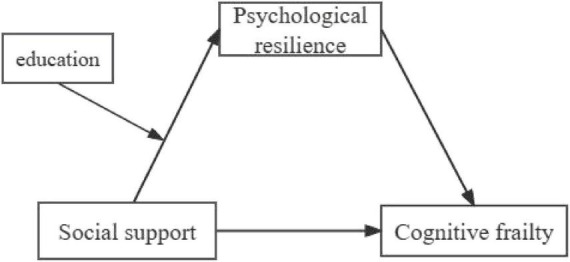
Theoretical model: a moderated mediation model.

From the above analysis, it is possible that PR may act as a mediator of the relationship between SS and CF, and education as a moderator of the relationship between SS and PR. However, to date, no studies have comprehensively explored the factors underlying the relationship between SS and CF among older adults. Therefore, the present study seeks to assess the prevalence of CF, explore whether PR mediates the association between SS and CF, and evaluate a moderated mediation model. In the moderated mediation model, we hypothesize that PR might work as a mediator between SS and CF among the older adults. Furthermore, education might play a role as a moderator in the indirect effect (path: SS–PR) of SS on CF (see Figure 1).

## 2 Materials and methods

### 2.1 Study design and sample selection

This cross-sectional survey was performed in Jinan, Shandong province, China from July to August in 2023. Based on the level of economic development, we adopted a stratified cluster sampling from twelve administrative. First, according to the GDP per capita in Jinan in 2022, Licheng (one above the medium level), Zhangqiu (one at the medium level), Pingyin (one below the medium level) three districts/counties were adopted. Second, two townships/streets are randomly selected from each district/county. Third, we randomly selected five communities/villages from each of selected townships/streets and the older adults aged over 60 years who were randomly selected from each sample communities/villages were recruited in the survey. The older adults with capability of verbal communicative, aged 60 and over were recruited in the study, and excluded patients with terminal illness, a history of mental illness or Parkinson’s disease, Alzheimer’s disease or other dementia, as well as older adults with severe visual or hearing impairment, physical disability, physical activity limitation due to physical trauma, and severe physical illness.

The sample size calculation was performed by the following for formula: $\text{n}=\frac{{\text{u}}_{\alpha }^{2}\,\pi \,\left(1-\pi \right)}{\delta^{2}}$ (π:expected prevalence) (Sun, 2002). The prevalence of incidence of CF in the older adults was *p* = 6.6% according to previous studies (Zhao et al., 2020)., so in the study of the current situation of the older adults with CF, π = 6.6%, u_α_ = 1.96, δ = 0.02, α = 0.05. We found that the required sample size was 593. The total sample size obtained in our study was 1,819, which has reached the required sample size. Therefore, our sample is representative and sufficient.

### 2.2 Data collection

All the respondents were interviewed face-to-face using a structured questionnaire by trained interviewers with medical knowledge. To obtain complete and accurate data, we excluded the respondents who could not answer independently and with a history of dementia by further asking the village doctors about the physical condition of the interviewees. A total of 1,819 older adults were recruited, of which 22 respondents were excluded from the analysis due to lack of key data and 39 respondents were excluded from the analysis due to being diagnosed with a disability by a physician. Finally, 1,758 respondents were included in the analysis. The 1,758 samples included and the 61 samples not included were balanced in basic characteristics (see Supplementary Table 1 for detail).

### 2.3 Measures

#### 2.3.1 Social support

The Social Support Rating Scale (SSRS) (Xiao, 1994) was used to measure SS. The SSRS is a 10-item self-reported scale composed of three parts: subjective support, objective support, and support utilization. Subjective support reflects perceived SS that individuals feel understood, supported, or helped by others. Objective support represents the actual support that individuals received, such as the financial support and the practical assistance. The utilization of support reflects the degree of SS used, such as individuals how to seek and get actual help when in need. The SSRS has been shown a reliability and validity measure in China (Jin et al., 2020). The Cronbach alpha index confirmed the acceptable internal consistency of SSRS in the study, with a value of 0.665. The total score of SSRS ranges from 12 to 66, with higher scores indicate higher levels of SS.

#### 2.3.2 Psychological resilience

Connor-Davidson Resilience Scale-10 item (CD-RISC-10) (Campbell-Sills and Stein, 2007) was applied to measure PR of the older adults. Each item is scored on a 5-point Likert scale (0 = never, 1 = rarely, 2 = sometimes, 3 = usually, 4 = always) and the total sum score ranges from 0 to 40, with higher scores reflecting better resilience. The CD-RISC-10 showed high internal consistency in this study (Cronbach’s α = 0.964).

#### 2.3.3 Cognitive frailty

According to the definitions provided by I.A.N.A and I.A.G.G, respondents who had the co-existence of physical frailty and cognitive impairment were classified as having CF in this study. The independent variable measurement was based on previous literature, using Fried frailty phenotype and Mini Mental State Examination (MMSE) (Shimada et al., 2013). Participants who tested positive on both assessments were classified as having CF and coded as “1,” the others were coded as “0.”

Physical frailty was measured by an adapted version of the Fried physical phenotype approach (Fried et al., 2001). Five items are included: shrinking, weakness, exhaustion, slowness, and low activity, as detail following:

Shrinking, unintentional body weight loss of ≥ 5% in prior year.

Weakness, the max of the three dominant hand grip strength test results after adjusting for BMI and gender. The criterion of male weakness: BMI ≤ 24.0 and grip strength < 29.0 kg; 24.1 ≤ BMI ≤ 26.0 and grip strength < 30.0 kg; 26.1 ≤ BMI ≤ 28.0 and grip strength < 30.0 kg; BMI > 28.0 and grip strength < 32.0 kg. The criterion of females weakness: BMI ≤ 23.0 and grip strength < 17.0 kg; 23.1 ≤ BMI ≤ 26.0 and grip strength < 17.3 kg; 26.1 ≤ BMI ≤ 29.0 and grip strength < 18.0 kg; BMI > 29.0 and grip strength < 21.0 kg.

Exhaustion, as indicated by two questions from the Center for Epidemiologic Studies-Depression scale. “I felt that everything I did was an effort”; “I could not get going.” Participants with a frequency of 3–7 days or 5–7 days a week were classified as exhaustion.

Slowness, determined by the time taken to walk 4.6-meters, adjusted for gender and standing height. For males: height ≤ 173 centimeter (cm) and speed ≥ 7 s; height > 173 cm and speed ≥ 6 s. For females: height ≤ 159 cm and speed ≥ 7 s; height > 159 cm and speed ≥ 6 s.

Low activity, evaluated by querying the following question about time spent engaged in sports and exercise “How often did you exercise in the 12 months before the survey?” Participants with a frequency of 1–3 a month or lesser were classified as low activity (Chen et al., 2020).

Participants who met three or more criteria were defined as having physical frailty (frail), otherwise, they were considered as having no physical frailty (nonfrail).

Cognitive impairment was assessed by the Chinese version of the Mini Mental Status Examination (MMSE) (Folstein et al., 1975). It includes the following seven dimensions: orientation to time, orientation to place, immediate memory, delayed memory, attention and computation, language and spatial vision. The 30-item MMSE score ranges from 0 to 30, with a higher score indicating a better cognition. MMSE has been widely used in the assessment of cognitive function level in older adults and has excellent reliability and validity (Fang et al., 2019). The Cronbach alpha index of the MMSE was 0.880, which has high internal consistency. The cut-off values of the MMSE for cognitive impairment according to educational level were ≤ 17 for the illiterate, ≤ 20 for the primary school educated, ≤ 22 for those accepted the junior high school, and ≤ 24 for those university or above, respectively (Katayama et al., 2021).

#### 2.3.4 Sociodemographic variables and other covariates

Demographic characteristic included gender (male, female), age (60∼, 70∼, ≥ 80), education (illiterate, primary school, middle school, high school or above), marital status (single, married), residence (urban, rural). Other covariates were identified for SS and CF based on the previous studies (Katayama et al., 2021; Xie et al., 2021; Yeh and Liu, 2003), those including number of chronic disease (that was divided into three categories: “zero,” “one,” and “two or more”), household income [that was divided into quartiles: the first quartile (Q1), the second quartile (Q2), the third quartile (Q3), and the fourth quartile (Q4)], cigarette smoking, alcohol drinking.

### 2.4 Statistical analysis

IBM SPSS version 26.0, Stata 16.0 and Mplus 8.3 were utilized for analysis. The reported confidence intervals were calculated at the 95% level and *P*-values less than 0.05 were considered statistically significant. Firstly, we used descriptive analyses to describe the demographics. Secondly, Mann–Whitney U tests and chi-square tests were employed to explore the differences between older adults with and without CF. Thirdly, Spearman coefficients were used to test the correlations between variables such as SS, PR, education, and CF. Fourthly, a mediation model suggested by Mackinnon and Dwyer (1993) and Wen and Ye (2014a) was used to test the mediating effect of PR on the relationship of SS and CF: (1) linear regression analysis was used to explore the association between SS and PR. (2) binary logistic regression was employed to test the association between SS and CF while including the mediator, PR. Fifthly, a moderated mediation model (Wen and Ye, 2014b; Ye and Wen, 2013) was used to test the moderated mediation effect of education on the relationship between SS and CF, with PR serving as a mediator: (1) linear regression analysis was conducted to explore the association between SS and PR, incorporating the moderator and interaction terms (social support × education) into this relationship. (2) binary logistic regression was employed to test the association between SS and CF, taking into account the mediator, PR. All sociodemographic characteristics, number of chronic disease, household income, cigarette smoking, alcohol drinking were controlled for in the above analyses. Additionally, Bootstrap analyses were conducted using 5,000 bootstrap samples and bias-corrected 95% confidence intervals. All continuous variables were centered.

Harman’s single factor test was employed in the study to uncover common method bias. This method is commonly used in approach bias testing (Podsakoff et al., 2003). Harman’s single-factor test showed that in the exploratory factor analysis, there were eleven factors with characteristic roots greater than one. The first principal component explained 22.78% of the total variance, which was lower than the critical value of 40% (Podsakoff et al., 2003). It indicated that there was no significant common method bias in this study.

### 2.5 Ethical consideration

This study was approved by the Medical Ethical Committee of the Jinan Center for Disease Control and Prevention, where the researcher worked (approval number, JIJILUNPI[2024]004; approval date, June 19, 2023). The investigation was conducted after the acquisition of informed consent of all participants.

## 3 Results

### 3.1 Characteristic of participants

The study included 1,758 participants and Table 1 shows the participants’ characteristics. Among the 1,758 older adults, 4.3% experienced CF. Participants had a median total SS score of 38 points, with an interquartile range of 9, and those with CF scored a median total of 34 points, with an interquartile range of 10.5. Participants had a median total score of 30 points for PR, with an interquartile range of 11, whereas those with CF scored a median of 20 points, with an interquartile range of 16. For those older adults, the majority of them were female (61.4%), under the age of 70 (46.8%), married (78.7%), with the education level of primary school (40.4%), residence of rural (87.3%), suffering from one chronic disease (40.6%), the household income was poorest (26.5%), did not smoke (88.3%), did not drink alcohol (77.2%). The study showed that age, education, marital status, number of chronic disease, residence, household income, SS and PR of the older adults with CF and without CF were statistically significant differences (*P* < 0.05). More details of the participants’ characteristics are shown in Table 1.

**TABLE 1:** Description and univariate analysis of cognitive frailty among older adults in Jinan, China, 2023 (*N* = 1,758).

Characteristics	*N* (%)	Cognitive frailty		X^2^/Z	*p*-value
		**No (%)**	**Yes (%)**		
Observations	1,758	1,682 (95.7)	76 (4.3)		
**Gender**				2.31	0.128
Male	678 (38.6)	655 (96.6)	23 (3.4)		
Female	1,080 (61.4)	1,027 (95.1)	53 (4.9)		
**Age**				46.12	< 0.001
60∼	822 (46.8)	803 (97.7)	19 (2.3)		
70∼	805 (45.8)	768 (95.4)	37 (4.6)		
≥ 80	131 (7.5)	111 (84.7)	20 (15.3)		
**Education**				79.06	< 0.001
Illiterate	402 (22.9)	353 (87.8)	49 (12.2)		
Primary school	711 (40.4)	694 (97.6)	17 (2.4)		
Middle school	475 (27.0)	466 (98.1)	9 (1.9)		
High school or above	170 (9.7)	169 (99.4)	1 (0.6)		
Marital status				4.97	0.026
Single*	375 (21.3)	351 (93.6)	24 (6.4)		
Married	1,383 (78.7)	1,331 (96.2)	52 (3.8)		
Number of chronic disease				13.02	< 0.01
0	494 (28.1)	486 (98.4)	8 (1.6)		
1	713 (40.6)	678 (95.1)	35 (4.9)		
≥ 2	551 (31.3)	518 (94.0)	33 (6.0)		
**Residence**				5.53	0.019
Rural	1,534 (87.3)	1,461 (95.2)	73 (4.8)		
Urban	224 (12.7)	221 (98.7)	3 (1.3)		
**Household income+**				45.83	< 0.001
Q1	465 (26.5)	421 (90.5)	44 (9.5)		
Q2	410 (23.3)	392 (95.6)	18 (4.4)		
Q3	447 (25.4)	441 (98.7)	6 (1.3)		
Q4	436 (24.8)	428 (98.2)	8 (1.8)		
**Cigarette smoking**				1.12	0.289
No	1,552 (88.3)	1,482 (95.5)	70 (4.5)		
Yes	206 (11.7)	200 (97.1)	6 (2.9)		
**Alcohol drinking**				3.135	0.077
No	1,357 (77.2)	1,292 (95.2)	65 (4.8)		
Yes	401 (22.8)	390 (97.0)	11 (2.7)		
Social support, M (P25, P75)	38 (33, 42)	38 (33, 42)	34 (27.5, 38)	−5.719	< 0.001
Psychological resilience, M (P25, P75)	30 (26, 37)	30 (27, 37)	20 (13, 29)	−8.865	< 0.001

*Single include those who are unmarried (26, 1.48%), divorced (342, 19.45%), widowed (5, 0.28%), and others (2, 0.11%); +Q1 was the poorest and Q4 was the richest.

### 3.2 Correlation between key variables

The correlation matrix for key study variables is provided in Table 2. Spearman’s correlation analysis revealed that there was a significant negative association between CF and SS (ρ = −0.136, *p* < 0.001). CF was negatively correlated with PR (ρ = −0.211, *p* < 0.001) and negatively related to education (ρ = −0.176, *p* < 0.001). SS was positively correlated with PR (ρ = 0.210, *p* < 0.001) and positively correlated to education (ρ = 0.170, *p* < 0.001). PR was positively correlated with education (ρ = 0.192, *p* < 0.001).

**TABLE 2:** Spearman correlation coefficients between key study variables (*N* = 1,758).

Variables	Social support	Psychological resilience	Education	Cognitive frailty
**Social support**	1			
Psychological resilience	0.210***	1		
Education	0.170***	0.192***	1	
Cognitive frailty	−0.136***	−0.211***	−0.176***	1

*p < 0.05; **p < 0.01; ***p < 0.001.

### 3.3 Mediation analysis

In this study, the bias-corrected percentile Bootstrap test was used to extract 5000 repetitions to calculate the 95% confidence interval. After adjusted for control variables, SS was significantly positively associated with PR (*B* = 0.272, 95% CI: 0.211, 0.333), and SS, PR was significantly negatively associated with CF (*B* = −0.066, 95% CI: −0.107, −0.026; *B* = −0.103, 95% CI: −0.129, −0.075) (see detail for Table 3). The mediation effect test of PR shows a significant direct effect of SS on CF; PR played a significant role in mediating the relationship between them. Specifically, the standardized total effect of SS on CF through PR was estimated as −0.258 (95% CI:−0.366, −0.160), the standardized direct effect was estimated as −0.181 (95% CI: −0.287, −0.086), and the standardized indirect effect was estimated as −0.077 (95% CI: −0.105, −0.051) (see detail for Table 4).

**TABLE 3:** Mediation effect of social support on cognitive frailty (by parallel mediation analysis).

Outcome variables	Psychological resilience		Cognitive frailty		
	***B***	**95% CI**	***B***	**OR**	**95% CI**
Social support	0.272***	(0.211, 0.333)	−0.066**	0.937	(−0.107, −0.026)
Psychological resilience			−0.103***	0.902	(−0.129, −0.075)

Controlling for gender, age, marital status, number of chronic disease, residence, household income, cigarette smoking, alcohol drinking.

*p < 0.05;

**p < 0.01;

***p < 0.001.

**TABLE 4:** The standardized total, direct, and indirect effects of social support on cognitive frailty with psychological resilience as mediators (*N* = 1,758).

Model pathways	*B*	SE	95% CI	Mediating effect
Total effect social support→cognitive frailty	−0.258***	0.053	(−0.366, −0.160)	100%
Direct effect social support→cognitive frailty	−0.181**	0.052	(−0.287, −0.086)	70.16%
Indirect effect social support→psychological resilience→cognitive frailty	−0.077***	0.014	(−0.105, −0.051)	29.84%

*p < 0.05; **p < 0.01; ***p < 0.001.

### 3.4 Moderated mediation analysis

Table 5 showed the results of moderated mediation. According to the hypothesis, education may function as a moderator between SS and PR. The results showed that education moderated the association between SS and PR. After controlling for covariates, the interaction effect of SS and education was found to be significant. Specifically, older adults with higher education level (including primary school, middle school, and high school or above) have significantly higher PR than the illiterate group. Furthermore, compared to the illiterate, with the increase of education level, the influence effect of SS on PR gradually weakened, which suggests that education may buffer the positive effect of SS on PR to some extent. The significant moderated mediation model was further tested by analyzing the indirect effect of SS on CF at different levels of education. As shown in Table 6, PR significantly mediated the association between SS and CF among the older adults with different educational backgrounds: illiterate (*B* = −0.044, 95% CI: −0.065, −0.029), primary school (*B* = −0.025, 95% CI: −0.037, −0.015), and middle school (*B* = −0.019, 95% CI: −0.032, −0.007). Importantly, it could be observed that the indirect effect of SS on CF through PR weakened with the increase of education level.

**TABLE 5:** Bootstrap confidence intervals for the moderated mediation effects.

Predictor	Psychological resilience		Cognitive frailty		
	***B***	**95% CI**	***B***	**OR**	**95% CI**
Social support	0.427***	(0.298, 0.558)	−0.066**	0.937	(−0.110, −0.028)
Psychological resilience			−0.103***	0.902	(−0.134, −0.079)
**Education**					
Illiterate	1				
Primary school	2.092***	(1.038, 3.172)			
Middle school	2.148**	(0.932, 3.339)			
High school or above	2.042*	(0.373, 3.713)			
Social support*education					
Social support*Illiterate	1				
Social support*primary school	−0.184*	(−0.331, −0.037)			
Social support*middle school	−0.244**	(−0.411, −0.079)			
Social support*high school or above	−0.315**	(−0.548, −0.089)			

Controlling for gender, age, marital status, number of chronic disease, residence, household income, cigarette smoking, alcohol drinking.

*p < 0.05;

**p < 0.01;

***p < 0.001.

**TABLE 6:** Indirect effect of social support on cognitive frailty moderated mediation by education.

Social support→psychological resilience→cognitive frailty			
**Condition (level of moderator -education)**	***B***	**SE**	**95% CI**
Illiterate	−0.044***	0.009	(−0.065, −0.029)
Primary school	−0.025***	0.006	(−0.037, −0.015)
Middle school	−0.019**	0.006	(−0.032, −0.007)
High school or above	−0.012	0.010	(−0.032, 0.008)

*p < 0.05;

**p < 0.01;

***p < 0.001.

**FIGURE 2:**
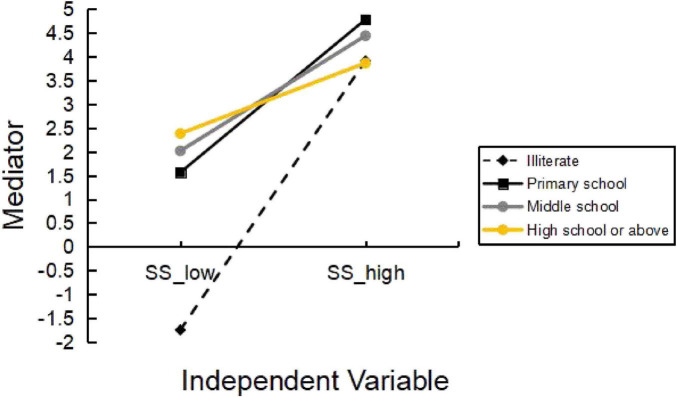
Moderation of education on SS and PR. SS, social support; PR, psychological resilience.

To better understand the moderating effect of education, a further simple slope analysis was conducted to distinguish between high and low levels of the independent variable by means plus or minus one standard deviation. Simple slope tests (Figure 2) showed that compared to illiterate (*B* = 0.427, *p* < 0.01), primary school (*B* = 0.243, *p* < 0.01), middle school (*B* = 0.183, *p* < 0.01) and high school or above (*B* = 0.112, *p* < 0.01) significantly attenuated the enhancing effect of SS on PR. Specifically, for the older adults with lower levels of education (such as illiterate and primary school), there was a more pronounced improvement in the levels of PR with increased SS. However, as educational attainment increased (especially reaching middle school or higher levels), the positive effect of SS on PR diminished. In essence, compared to lower levels of education, higher levels of education may weaken the associations between SS and PR.

## 4 Discussion

This study investigated the prevalence of CF, examined the mediating role of PR between SS and CF, and explored the relationships among SS, education, PR and CF via a moderated mediation model. The findings indicated that SS, education and PR were all significantly correlated to CF, but the effects of these factors were different. PR mediated the association between SS and CF. Education moderates the association between SS and PR. These findings help elucidate the underlying causes of CF and facilitate the development of targeted interventions, so as stop or delay the progression of CF in the older adults.

In the present study, the prevalence of CF among the older adults was 4.3%, lower than in urban Henan, China (9.6%) (Pan et al., 2019) and Brazil (10.9%) (Brigola et al., 2020) of the older adults, but higher than community dwelling older adults in China (3.3%) (Ma et al., 2017) and Japan (2.7%) (Shimada et al., 2013). Recent studies show CF rates ranging from 1.2% to 39.6% (Rivan et al., 2021; Shimada et al., 2016), with discrepancies due to regional differences, varied measurements, and sample age structures. SS significantly predicted CF, with higher SS levels associated with a lower likelihood of CF, which is consistent with the previous research (Malek Rivan et al., 2019).

Our findings showed that PR mediated the association between SS and CF, with the mediating effect accounted for 29.84%. The great SS available to the older adults, the higher their PR and the lower the incidence of CF. This finding is in accordance with previous research revealing that increased levels of SS are associated with increased higher levels of PR (Liao et al., 2022), which is considered to be a protective factor against the likelihood of older adults entering vulnerable states (Lenti et al., 2022). The ample SS received by the older adults provides them with rich emotional support, sense of belonging and access to resources, and bolstering their ability to adapt and cope with stress (Cohen and Wills, 1985), thereby enhancing PR (Lenti et al., 2022). Researchers believe that the older adults with higher PR are more likely to maintain a positive outlook and employ effective coping strategies when faced with health challenges, such as illness or declining physical function (Li et al., 2021). This positive coping style helps to alleviate the impact of the frailty state on daily functioning. This is consistent with the framework of PR proposed by Kumpfer (1999), that is, external resources (such as SS) need to be transformed into health outcomes through individual internal capabilities (such as PR).

The effect of education on the association between SS and PR was explored in this research. The study revealed that education moderated the association between SS and PR in older adults. The older adults with varying levels of education exhibited different trends in PR in response to varying levels of SS. Specifically, present study showed that the older adults in the lowest education level (illiterate) group exhibited the lowest levels of PR when the level of SS was low, and their PR significantly increased with the increase of SS, and the increase was the largest. However, with the increase of the education level of the older adults, the positive effect of SS on PR gradually weakened. These findings echo previous research: older adults with higher education levels usually have stronger cognitive reserve and social resource access, which may partially substitute for the effect of SS on PR (Chen and Feeley, 2014; Mirowsky, 2003). In the face of stressful events, the older adults with low education level rely more on external support to cope with stress (Mirowsky, 2003), while the older adults with high education level tend to combine external support with internal resources to further enhance their internal literacy and reduce their dependence on external resources (Mirowsky, 2003; Niemeyer et al., 2019). That is, education level, as an indicator of socioeconomic status, may affect an individual’s ability to access and utilize social resources (Zhang et al., 2022), thus acting as a moderator between SS and PR. Notably, in addition to the moderating effect of education, education may directly contribute to reducing CF in older adults by enhancing cognitive reserve (Livingston et al., 2020; Stern, 2002). Higher education levels are associated with stronger synaptic density and neural efficiency, thus buffering CF (Arenaza-Urquijo and Vemuri, 2018; Bartrés-Faz and Arenaza-Urquijo, 2011). The heterogeneous effect of educational stratification suggests that education level should be included in the CF screening system for the older adults, and designing personalized aging intervention for educational differences is the key strategy (Brigola et al., 2019; Zahodne et al., 2015).

The current study had several limitations concerning the interpretation of results. First, given the cross-sectional nature of the data, we could not examine the direction of the causal relationship among SS and CF. Future longitudinal studies should be conducted to address this issue and explore underlying mechanisms in more details. Second, the participants in our study were limited to one location, and it is unclear whether these findings will generalize beyond this sub-sample of the population. Third, there may be other potential mechanisms to be further explored in the study of the relationship between SS and CF. Future studies will explore more potential pathways to comprehensively explain the association between SS and CF, drawing on relevant theories frameworks and empirical evidence.

## 5 Conclusion

The prevalence of CF was 4.3% among older adults. Our findings revealed that association between SS and CF, in which PR mediates this relationship and education plays an key moderating role in the relationship between SS and PR. Especially for the older adults with lower levels of education, increasing the level of SS may help to significantly improve their PR and reduce the incidence of CF. Therefore, based on the above mechanism, this study proposes to construct a three-level hierarchical intervention system. Specifically, at the community level, the SS level of the older adults is improved by means of neighborhood help and social activities, especially to provide targeted support environment for the older adults with low education level; at the medical level, the network platform was used to provide mental health education and cognitive training courses, and the level of PR was improved through accessible digital intervention; at the family-society level, an inter-generational mutual assistance model was constructed, and a health protection buffer was formed through emotional support and the construction of an aging environment. The system provides a comprehensive support network for the older adults by synergized strengthening the association of SS→PR→CF pathway, and provides evidence-based basis for CF prevention and control and aging health promotion.

## Data Availability

The original contributions presented in this study are included in this article/Supplementary material, further inquiries can be directed to the corresponding authors.
