# Supplementary material for: Effects of social support on cognitive frailty among the older adults in China: mediation of psychological resilience and moderated mediation of education
Source: Front Aging Neurosci. 2025 May 16;17:1579485. doi: 10.3389/fnagi.2025.1579485 (PMC12123642; doi:10.3389/fnagi.2025.1579485)
Supplement: Supplementary file 1 [file Table_1.docx]

Supplementary Material

Table 1 Comparison of basic characteristics between included samples (n=1758) and excluded samples (n=61)

| Characteristics | Included(n=1758) | excluded(n=61) | p-value |
| --- | --- | --- | --- |
| Gender |  |  | 0.157 |
| Male | 678(38.6%) | 29(47.5%) |  |
| Female | 1080(61.4%) | 32(52.5%) |  |
| Age |  |  | 0.092 |
| 60~ | 822(46.8%) | 24(39.3%) |  |
| 70~ | 805(45.8%) | 28(45.9%) |  |
| ≥80 | 131(7.4%) | 9(14.8%) |  |
| Education |  |  | 0.156 |
| Illiterate | 402(22.9%) | 19(31.1%) |  |
| Primary school | 711(40.4%) | 27(44.3%) |  |
| Middle school | 475(27.0%) | 13(21.3%) |  |
| High school or above | 170(9.7%) | 2(3.3%) |  |
| Marital status |  |  | 0.222 |
| Single* | 375(21.3%) | 17(27.9%) |  |
| Married | 1383(78.7%) | 44(72.1%) |  |
| Number of chronic  disease |  |  | 0.845 |
| 0 | 494(28.1%) | 16(26.2%) |  |
| 1 | 713(40.6%) | 27(44.3%) |  |
| ≥2 | 551(31.3%) | 18(29.5%) |  |
| Residence |  |  | 0.293 |
| Rural | 1534(87.3%) | 56(91.8%) |  |
| Urban | 224(12.7%) | 5(8.2%) |  |
| Household income+ |  |  | 0.121 |
| Q1 | 465(26.4%) | 22(36.1%) |  |
| Q2 | 413(23.5%) | 10(16.4%) |  |
| Q3 | 444(25.3%) | 19(31.1%) |  |
| Q4 | 436(24.8%) | 10(16.4%) |  |
| Cigarette smoking |  |  | 0.135 |
| No | 1552(88.3%) | 50(82.0%) |  |
| Yes | 206(11.7%) | 11(18.0%) |  |
| Alcohol drinking |  |  | 0.532 |
| No | 1357(77.2%) | 45(73.8%) |  |
| Yes | 401(22.8%) | 16(26.2%) |  |

Notes:

^*^Single include those who are unmarried, divorced, widowed, and others;

+Q1 was the poorest and Q4 was the richest.
